# Experimental taphonomy of fish - role of elevated pressure, salinity and pH

**DOI:** 10.1038/s41598-020-64651-8

**Published:** 2020-05-12

**Authors:** Fabian Gäb, Chris Ballhaus, Eva Stinnesbeck, Anna Gabriele Kral, Kathrin Janssen, Gabriele Bierbaum

**Affiliations:** 1 Institut für Geowissenschaften, University of Bonn, 53115 Bonn, Germany; 2 Institut für Medizinische Mikrobiologie, Immunologie und Parasitologie, University Hospital Bonn, 53127 Bonn, Germany

**Keywords:** Palaeontology, Geochemistry, Petrology

## Abstract

Experiments are reported to reconstruct the taphonomic pathways of fish toward fossilisation. Acrylic glass autoclaves were designed that allow experiments to be carried out at elevated pressure up to 11 bar, corresponding to water depths of 110 m. Parameters controlled or monitored during decay reactions are pressure, salinity, proton activities (pH), electrochemical potentials (Eh), and bacterial populations. The most effective environmental parameters to delay or prevent putrefaction before a fish carcass is embedded in sediment are (1) a hydrostatic pressure in the water column high enough that a fish carcass may sink to the bottom sediment, (2) hypersaline conditions well above seawater salinity, and (3) a high pH to suppress the reproduction rate of bacteria. Anoxia, commonly assumed to be the key parameter for excellent preservation, is important in keeping the bottom sediment clear of scavengers but it does not seem to slow down or prevent putrefaction. We apply our results to the world-famous Konservat-Lagerstätten Eichstätt-Solnhofen, Green River, and Messel where fish are prominent fossils, and reconstruct from the sedimentary records the environmental conditions that may have promoted preservation. For Eichstätt-Solnhofen an essential factor may have been hypersaline conditions. Waters of the Green River lakes were at times highly alkaline and hypersaline because the lake stratigraphy includes horizons rich in sodium carbonate and halite. In the Messel lake sediments some fossiliferous horizons are rich in FeCO_3_ siderite, a mineral indicating highly reduced conditions and a high pH.

## Introduction

**Figure 1:**
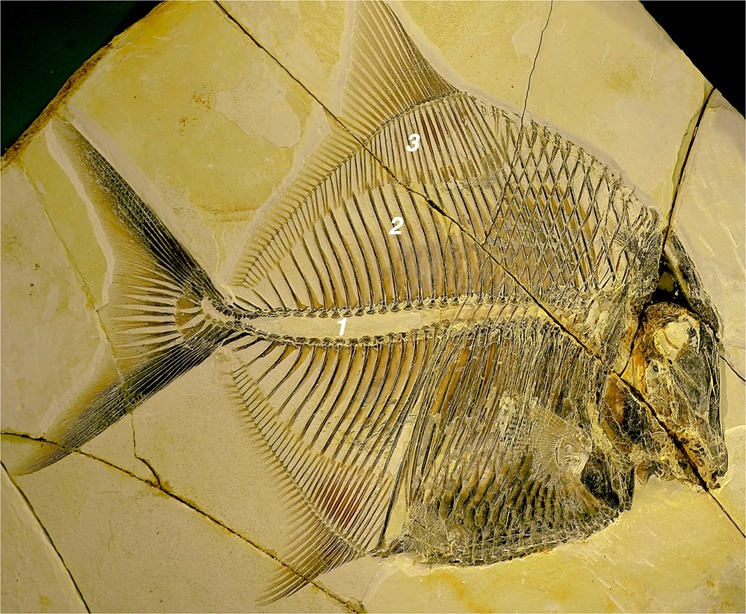
Delicate preservation of an Upper Tithonian Pycnodontid (*Proscinetes elegans*),  a Jurassic marine bony fish from the lithographic limestones of Ettling, Frankonian Alb (Ebert^49^). Vertebrae (1) of the genus *Proscinetes* not ossified (due to persistent notochord). Neural spines (2) well articulated with the pterygiophores of the dorsal fin (3) even though this fossil is 150 Ma old. Collection number JME-ETT876, photograph with permission by the Jura-Museum Eichstätt.

Since the advent of experimental methods in paeontological research, our understanding of taphonomic and fossilisation reactions has much improved. Today we realise how easily and rapidly organic tissue may be transformed into inorganic materials^1–6^. Consensus is emerging that fossilisation reactions can take place within time frames accessible with laboratory experiments^7^. The near-perfect articulation of the Pycnodontid in Fig. 1 suggests that the decision for or against preservation must have been made early, shortly after the fish died. Under ambient marine conditions - oxygenated water, normal marine salinity, and near-neutral pH - a fish so delicate would have been disarticulated or consumed by scavengers within hours to days.

But what are the environmental factors most effective in retarding or preventing organic decay? If we identify those variables experimentally, we may hold the key to understanding the genesis of Konservat-Lagerstätten within which fish are prominent fossils. We report novel decay experiments with fish to understand early taphonomic pathways toward fossilisation. Parameters investigated experimentally are elevated hydrostatic pressure, elevated salinity, the role of proton (pH) and electron activity (Eh), bacterial activity, and time. We apply our results to three prominent Konservat-Lagerstätten where fossil fish are prominent species - Eichstätt-Solnhofen, Green River, and Messel.

### Previous taphonomic experiments

Our experiments build upon a number of pioneering taphonomic studies that have made important contributions to the mineralisation of organic tissue^2,8–11^. However, not all of these studies exerted strict control over the experimental parameters. Quite often, reaction containers were not sealed^8,12^ but then the pH of experimental solutions may be affected by ingression of atmospheric CO_2_. Redox states are often derived by measuring directly physically dissolved oxygen (O_2_,aq) with Clarke electrodes^1,2,12^ but at reduced conditions O_2_,aq is a poor redox proxy. For example, in equilibrium with iron sulfide at 25 °C^12^ the O_2_,aq is ~ 10^–68^ mol kg^−1^ and far too small to be quantified by direct measurement. To our knowledge, experiments at elevated salinities have not been conducted although hypersaline conditions are by no means rare in shallow epicontinental seas^13,14^. Several studies have emphasised the importance of bacterial mats in early fossilisation^8–10,15,16^, and consensus is emerging that the encrustration and lithification of a carcass by bacteria may counteract disarticulation. The only experimental study at elevated pressure was conducted by Saitta *et al*.^17^. These authors heated and pressed feathers and lizards to 250 °C and 300 bar to test if melanosomes in organic tissue may survive diagenesis.

### Experimental and analytical methods

All trials reported here were performed at elevated pressures. For that purpose we designed autoclaves that can simulate water depths of up to 110 m (11 bar). The autoclaves are machined from 110 mm diameter acrylic glass rods and permit organic decay reactions to be monitored optically as reactions proceed. Inner diameters are 70 mm, wall thicknesses are 40 mm, and the capacities are 570 cm^3^. Typical filling levels were around 450 cm^3^ to leave space for a gas cushion. Hydrostatic pressure was imposed by pressurising the autoclaves with N_2_ gas. Temperatures were kept at 22 ± 1 °C.

Experimental solutions used in the decay experiments were seawater and seawater brines with 3.5, 7, 10, and 14 wt.% NaCl equiv. Salinities were adjusted to the desired ionic strengths by evaporating Atlantic seawater. Most trials were carried out with goldfish *(Carassius auratus)* carcasses while a few experiments used cichlids of the genus and species *Thorichthys meeki* when goldfish specimens were unavailable. Prior to experimentation, all fish were euthanised with tricaine methanesulfonate (courtesy J. Mogdans, Zoology Department, University of Bonn). The sediment substrates onto which the carcasses were bedded were ultra-fine calcite (CaCO_3_) oozes with grain sizes of 3.5 ± 1 µm, and in two cases sodium acetate NaCH_3_COO and natron Na_2_CO_3_ * 10H_2_O. The latter two runs were performed to quantify how elevated pH levels affect the reproduction rates of bacteria and the decay rates of organic tissue.

Not only did the autoclaves serve as reaction containers. They were also used to calibrate the minimum pressure at which a fish carcass can sink to the bottom sediment. Fish that die at shallow depth usually surface when they have swim bladders, and are thus exposed to rapid decay. For a fish carcass to sink, a minimum water depth is required, deep enough to compensate for the buoyancy of the swim bladder. We have calibrated as a function of salinity with 14 goldfish specimens the hydrostatic pressures at which the carcasses reached neutral buoyancy. Neutral buoyancy is given when P = ρ * g * h, where P = hydrostatic pressure, ρ = density (salinity) of the solution, g = Earth’s acceleration, and h = height of the water column. The resulting function is highly non-linear. The curve was calibrated to 8 bar. It covers a salinity range from zero to 11.2 wt.% NaCl equiv.

Proton activities (pH) of the experimental solutions were recorded with Ag/AgCl hydrogen ion sensitive glass electrodes (EGA 151, Meinsberg; Gäb *et al*.^18^). The electrodes measure the electromotive force (emf) against the Ag-AgCl equilibrium and were calibrated with Merck standard solutions at pH 7 and 10. The nominal precision of the pH measurements was around ±0.1 units in pH. Electrochemical potentials (Eh, in mV) were monitored with Ag/AgCl combination glass electrodes and quantified against the potential of an internal Ag/AgCl reference immersed in 3n KCl solution. The pH and Eh measurements could only be performed prior to and after completion of an experiment since the glass electrodes cannot withstand the pressure gradient between ambient (1 atm) and experimental pressure (up to 8 bar). Replicate measurements suggest that Eh values are reproducible to within ±30 mV.

Goldfish carcasses that were physically preserved after the end of an experiment were dissected to document and compare the state of their organs with organs of a fresh (unreacted) goldfish. One specimen fermented in a hypersaline brine at 14 wt.% NaCl equiv. was scanned using a high-resolution SkyScan 1272 Bruker desktop micro computer tomograph (µCT). Articulation of its bones was imaged at an isotropic voxel resolution of 10 µm, using the Bruker software 3D.SUITE. With few exceptions, organs could not be imaged because density contrasts proved too low.

Bacteria populations of three experimental solutions were characterised by sequencing the 16 S rRNA genes. The microbiome analyses were carried out using the facilities of MR DNA in Texas^19^. We identify with this technique only the most abundant bacterial orders. A limitation is that 16 S rRNA sequencing does not allow to quantify absolute abundances of bacteria, nor does it permit a distinction to be made between the DNA of living and dead bacteria. Hence, the relative abundances of bacterial orders should be seen as a semi-quantitative only snapshot of all DNA that could be sequenced.

## Results

### Influence of pressure

**Figure 2:**
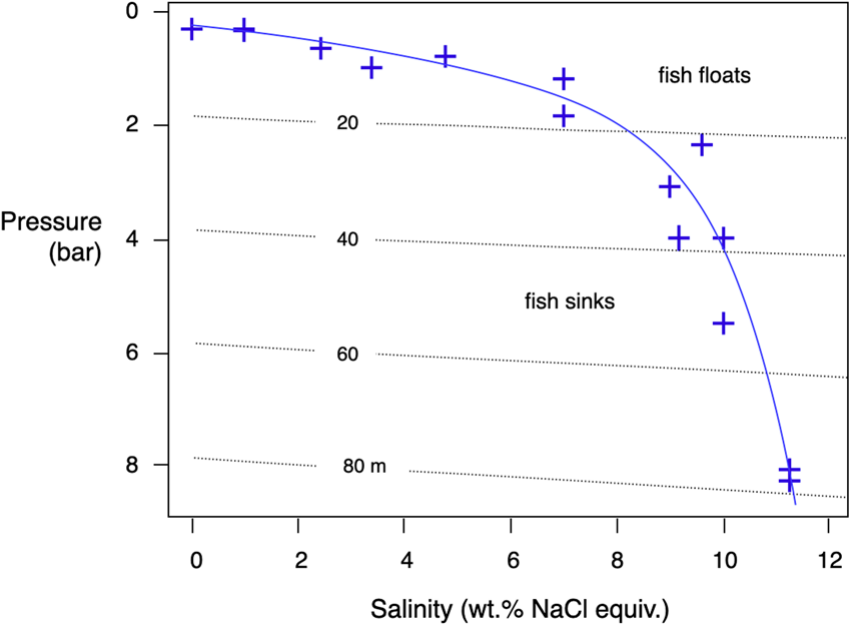
Neutral buoyancy curve calibrated with goldfish carcasses. The curve was calibrated at 22 ± 1 °C with variable salinities. Isobaths in meters calculated with P = ρ * g * h, where P = pressure, ρ = density, g = Earth’s acceleration, and h = height of water column.

Water depth is a key parameter for the fossilisation of fish. For fish to be successfully fossilised, they must sink after their death to be later be covered by sediment. We have calibrated as a function of water salinity the pressure at which a fish carcasses reaches its neutral buoyancy level (Fig. 2).

The neutral buoyancy curve is individual for goldfish with sizes that they fit the autoclave. Nonetheless, it is of general applicability:

- Fish dying at depths above the neutral buoyancy curve will float; such specimens would have little chance to be fossilised because they would rot quickly and/or be consumed by scavengers^20,21^.
- Fish dying at pressures higher than the neutral buoyancy curve will sink; they have the potential to be fossilised as soon as they are covered by sediment.
- The higher the salinity of the water, the higher the minimum pressure necessary for a fish carcass to sink, since water density increases with salinity. In basins stratified with respect to salinity the depth of the halocline and the salinity of the bottom waters will decide if a sinking carcass may reach the bottom sediment or if it will stagnate at the halocline.
- The larger the fish carcass, the higher the pressure necessary at given salinity that it may sink. Larger fish have a smaller surface-to-volume ratio, hence require higher pressures to experience the same percentage of compression as small fish.

Temperature also affects buoyancy because water density is temperature sensitive^22^ but in our pressure calibration experiments temperature was kept constant at 22 ± 1 °C.

**Figure 3:**
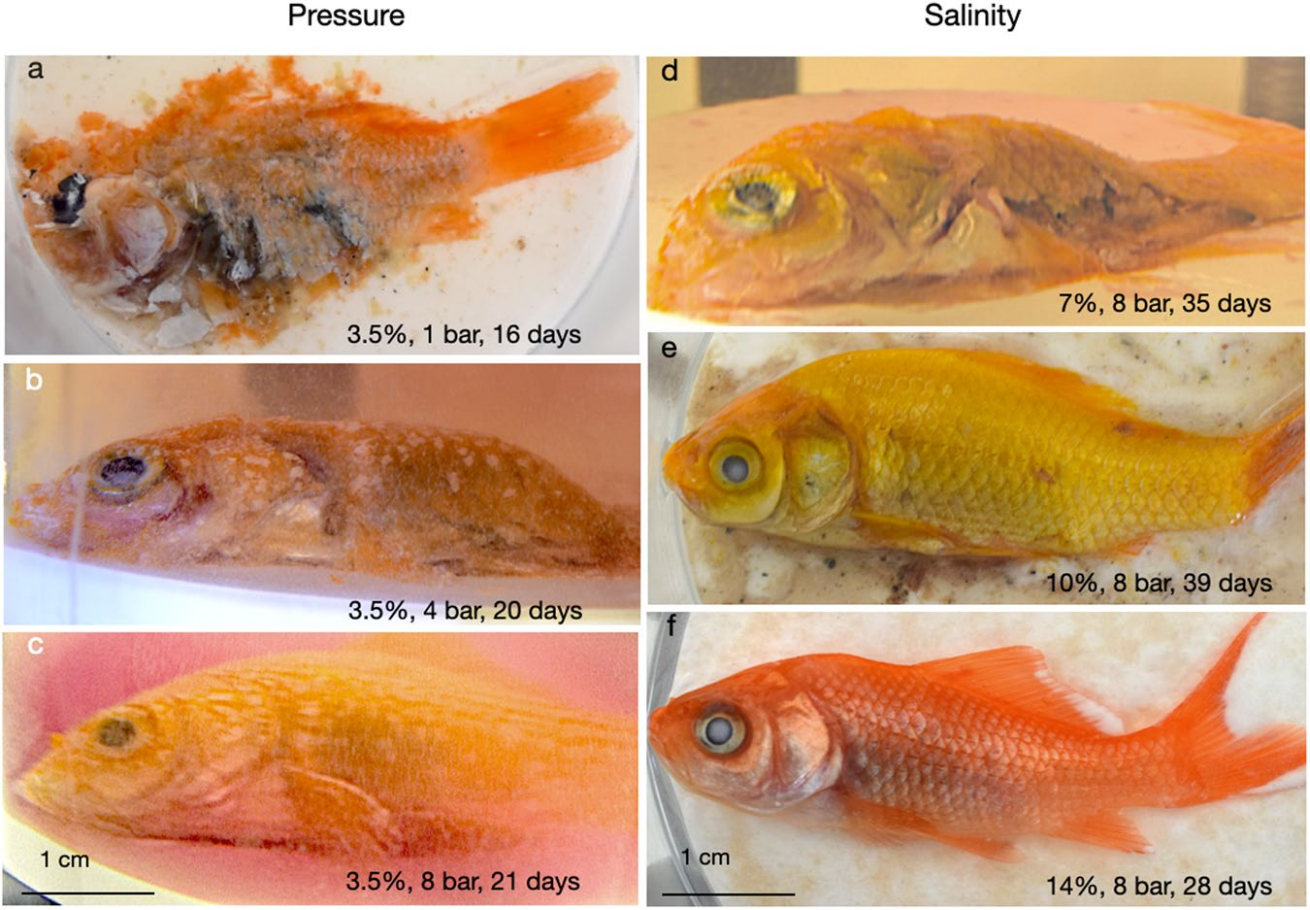
Influence of pressure and salinity on the preservation state of goldfish carcasses. The fish in (**b**–**d**) photographed *in situ* through the walls of the autoclave before decompression, (**a,e**,**f**) *ex situ* after decompression. Salt concentrations in wt.% NaCl equiv. of seawater brines.

Elevated pressure also helps to preserve fish carcasses externally (Fig. 3a-c). At pressures of 1 bar, disarticulation was noted within days. Fish treated at 4 and 8 bar were physically preserved but decomposed internally. Elevated pressure does not prevent putrefaction, however, it does seem to preserve the entity of a carcass by suppressing expansion and disruption by putrefaction gases.

### Influence of salinity

**Figure 4:**
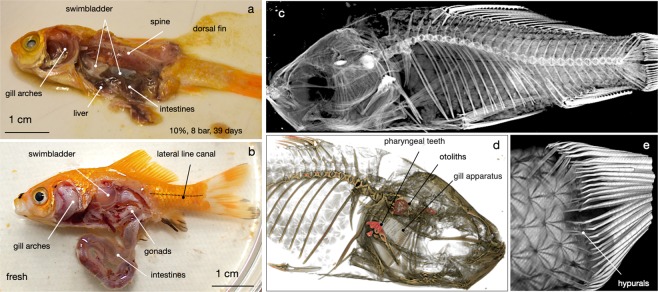
Preservation states of fish cured at high salinity. In Fig. 4a,b, organs of a 10% NaCl fish shown in Fig. 3e compared with organs of a freshly euthanised goldfish. In Figs. 4c to 4e, the 14 wt.% NaCl specimen shown in Fig. 3f scanned using a high-resolution desktop µCT system (SkyScan 1272, Bruker microCT) at an isotropic voxel resolution of 10 µm.

In Fig. 3d-f we show the conservation status of goldfish carcasses reacted in 3.5, 7, 10, and 14 wt.% NaCl equiv. brines. The specimen reacted in normal seawater (Fig. 3d) was found completely disarticulated and decomposed but the higher salinity specimens in Fig. 3e-f were found preserved externally. The fish fermented at 7 wt.% NaCl equiv. was ruptured ventrally but morphologically it was still intact. The two specimens in the 10 and 14 wt.% NaCl brines were well preserved both morphologically and internally. In Fig. 4a-b we compare the organs of the 10 wt.% NaCl carcass with organs of an unreacted goldfish carcass. The comparison shows that even after 39 days in 10 wt.% brine, organs are still allocable to their anatomic positions and rather well preserved.

The fish fermented at 14 wt.% NaCl was X-rayed and imaged with µCT (Fig. 4c-e). Articulation is perfect, all bones were found in place. In the scull, gill branches are still recognisable, and even the hypurals attached to the fin-rays, likely to be vulnerable to decay, are well preserved. A salt concentration of >10 wt.% NaCl equiv. seems to suppress the growth of decomposing micro-organisms so effectively that hardly any degradation is noted.

### Proton activity (pH) and electrochemical potential (Eh)

**Figure 5:**
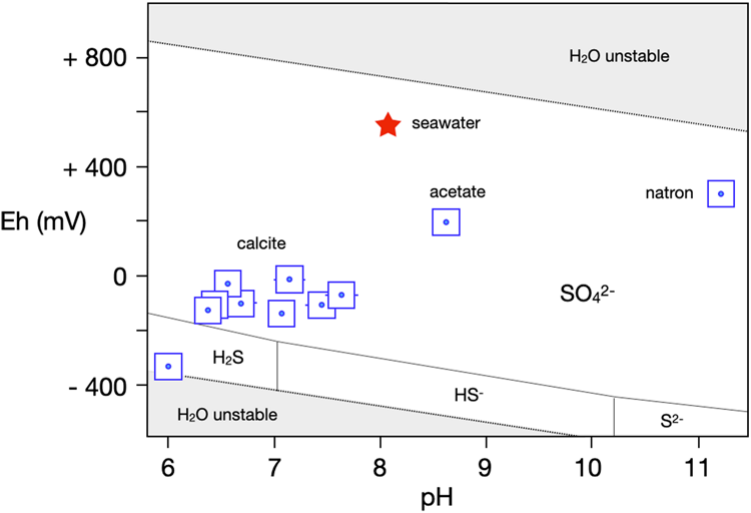
pH-Eh (Pourbaix) diagram. The graph illustrates the pH-redox relations of post-experiment solutions. Fish bedded on calcite CaCO_3_, Na-acetate NaCH_3_COO, or natron Na_2_CO_3_ * 10H_2_O. The trend of relative oxidation with increasing pH is taken to indicate that bacterial activity is suppressed as pH increases.

In Fig. 5 we display the pH-Eh conditions of all post-experiment solutions, recorded after the experiments were completed and the autoclaves opened. We display pH and Eh in one diagram to appreciate that protons and electrons are correlated through the equilibrium 6H_2_O = O_2_ + 4H_3_O^+^ + 4e^−^. Whenever CaCO_3_ was used as bottom sediment, the pH fell with reaction time from ~ 8.1 (seawater and seawater brine) to ~7 ± 0.5. Apparently, the decay of organic tissue liberates organic acids that hydrolyse with H_2_O to produce H_3_O^+^. Calcite as bottom sediment does not seem to have sufficient buffering capacity to neutralise oxonium at a rate acidity is released by organic decay.

To extend the pH range, two additional experiments were carried out with sodium acetate CH_3_COONa and natron Na_2_CO_3_*10H_2_O as bottom substrates. Both Na-acetate and natron are more soluble in water than CaCO_3_, both are more alkaline (pH ~ 9 and 11.2 respectively), and owing to their high solubilities in water they are more capable than CaCO_3_ to buffer pH. Indeed, the pH values of those two experiments ended up close to the saturation pH values of CH_3_COONa and Na_2_CO_3_*10H_2_O, at 8.6 and 11.2.

**Figure 6:**
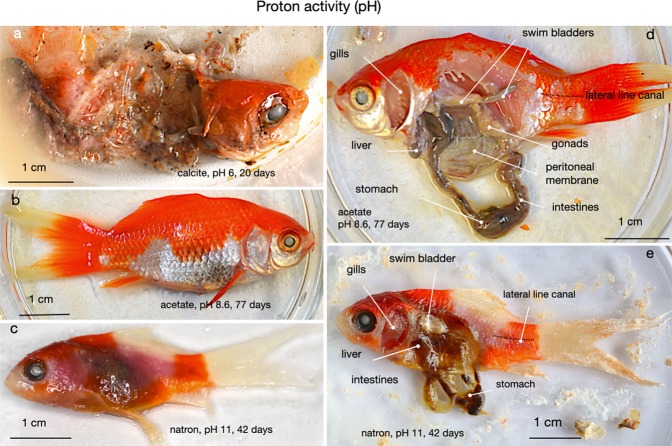
The influence of pH on the preservation state of fish. Figure 6a - a fish reacted on CaCO_3_ sediment (pH ~ 6.5 ± 0.5); Fig. 6b - a fish reacted on Na-acetate substrate (pH ~ 9); Fig. 6c - a fish reacted on a natron bed (pH = 11). In Fig. 6d,e, the acetate and natron fish are shown dissected to illustrate the preservation states of their organs.

With respect to electrochemical potentials, all experiments except one at a pH of 6 fell inside sulfate stability. It is noticeable that with increasing pH the solutions became more oxidized in pH-Eh space. Thermodynamically this trend is counter-intuitive. The decay of organic tissue given e.g. by

1 $${{\rm{C}}}_{6}{{\rm{H}}}_{12}{{\rm{O}}}_{6}\,({\rm{organic}}\,{\rm{tissue}})+24{{\rm{H}}}_{2}{\rm{O}}\to 6{{{\rm{HCO}}}_{3}}^{-}+18{{\rm{H}}}_{3}{{\rm{O}}}^{+}+12{{\rm{e}}}^{-}$$

produces oxonium ions as well as electrons, hence pH and Eh should be inversely correlated. This does not seem to be the case in Fig. 5. Our explanation is that alkaline conditions suppress bacterially mediated decay so effectively that no relative reduction occurs. Indeed, the fish on CaCO_3_ sediment at a pH of ~6 (Fig. 6a) was disarticulated after 20 days, while both the high-pH fish fermented on Na-acetate and natron beds (Fig. 6b-e) for 42 and 77 days were largely intact.

### Bacterial communities

Many, if not all early fossilisation reactions commence with carcasses being incrustrated by bacteria^15,16^. Iniesto *et al*.^10^ showed that fossils encased by bacterial mats are less susceptible to disarticulation. We also observed that after a few days in solution the carcasses were covered by red precipitates. Many experimental solutions turned out to be distinctly reddish (e.g. Figure 3b,c) notably those from experiments with normal-salinity seawater.

**Figure 7:**
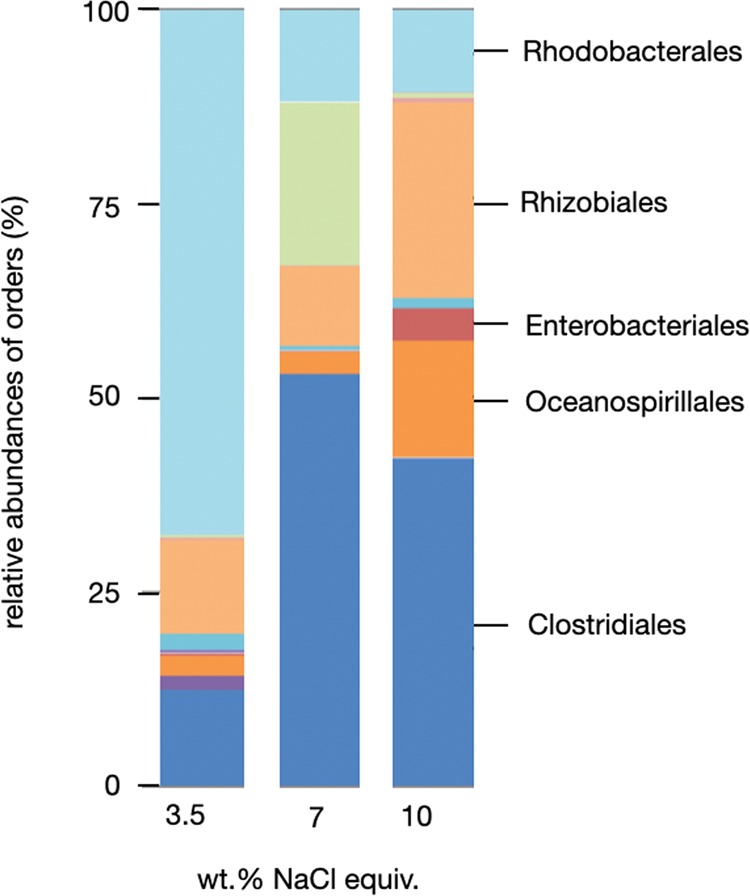
Relative abundances of bacterial orders. Bacterial orders identified by 16S rRNA sequencing for three post-experiment solutions with 3.5, 7, and 10 wt.% NaCl equiv.

To identify the principal bacteria populations, three experimental solutions with 3.5, 7, and 10 wt.% NaCl equiv. were sampled and the 16S rRNA genes were sequenced for microbiome analysis (Fig. 7). Well represented in the low salinity (3.5 wt.% NaCl) solution is the order *Rhodobacterales*. Species of that order perform anoxygenic photosynthesis and produce pigments that we may see in Fig. 3c. *Rhodobacterales* are key biofilm formers on surfaces in marine habitats^23^. Genera of the order *Rhizobiales* are nitrogen-fixing^24^ and apparently halotolerant since they were found most abundant in the 10 wt.% brine. Genera of the order *Oceanospirillales* are facultatively aerobic, some anaerobic, and some require elevated Na^+^ for their metabolism. The order *Clostridiales* includes alkaliphilic anaerobic spore forming bacteria that live in soils while many species of *Enterobacteriales* inhabit intestines. Overall, most bacterial orders identified here are anaerobic or facultatively anaerobic, an observation that accords well with generally low Eh measured in the three post-experiment solutions sequenced for RNA (Fig. 5).

### Burial by sediment

Fish can only be preserved through geologic time if they are covered by sediment. To simulate that situation, another experiment was performed where the fish carcass was fully embedded in calcite ooze The fish species used in this trial was a cichlid of the genus and species *Thorichthys meeki*. The water body above, and the pore solution inside the sediment, was seawater brine evaporated to 10 wt.% NaCl equiv. Hydrostatic pressure was 8 bar.

**Figure 8:**
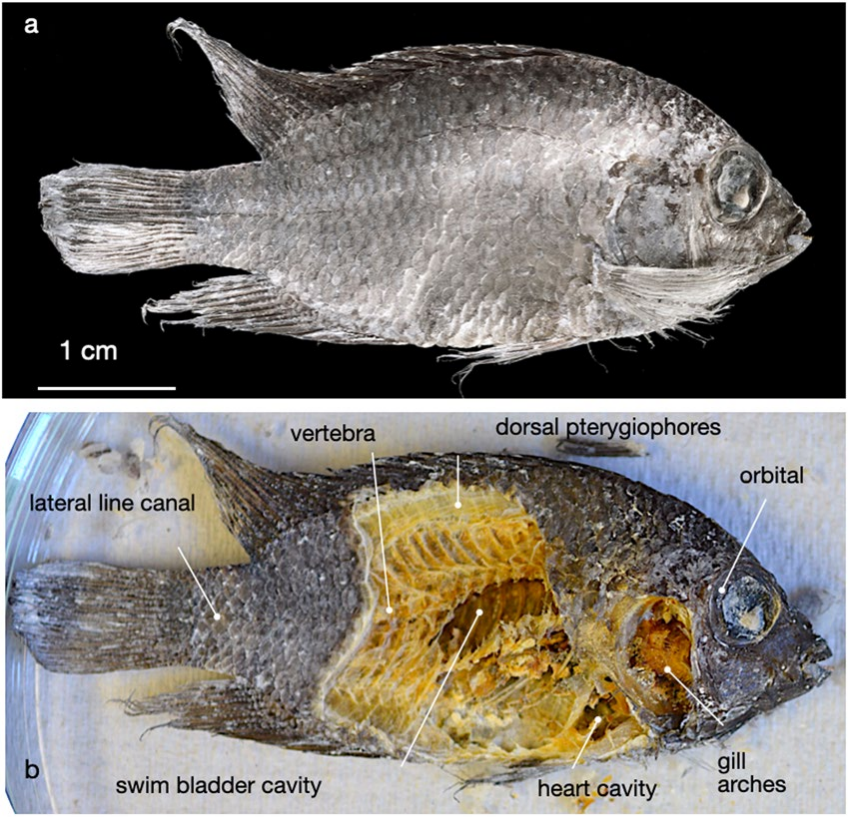
Fermentation of a fish carcass inside sediment. A cichlid (*Thorichthys meeki*) fermented inside CaCO_3_ ooze for 77 days, at a hydrostatic pressure of 8 bar in 10 wt.% NaCl equiv. pore water. In Fig. 8a the specimen after removal from the autoclave, in Fig. 8b, the same specimen dissected to illustrate the degree of articulation.

After 77 days, the cichlid was found flattened to ~ 5 mm thickness (Fig. 8a), presumably by a combination of osmotic dehydration and compaction. With respect to articulation, that specimen was found almost perfectly preserved. Internal organs are decomposed but their former anatomic positions still are readily identified (Fig. 8b). That experiment again highlights how effective high salinity may be for long-term preservation.

## Discussion

It is the initial conditions that determine the fate of a fish carcass, whether it decays or is handed down in the geological record as a well articulated fossil. The three parameters most effective in preventing decay are elevated salinity, elevated pH, and a hydrostatic pressure large enough that a fish after its death may sink to the bottom sediment.

An elevated salinity is highly effective in suppressing organic decay. That is not surprising; salt curing of food stuff has been used by humankind at least since 3000 BC^25^. High salt concentrations generate an osmotic pressure between cell membranes and the surrounding solution, thus causing bacteria to dehydrate. Accordingly, at salinities exceeding 10 wt.% NaCl equiv. little degradation of organic tissue was noted. Organs survived almost intact even after for 39 days (Fig. 4a). In the cichlid in Fig. 8a,b, fermented at identical salinity for 77 days, the organs are largely resorbed but all bones are almost perfectly articulated. Whether this is due to the fact that this specimen was fermented inside a carbonate matrix or dried before it was dissected cannot be verified. It is clear though that the 77 day cichlid would have had excellent chances to “survive” as a fossil as well articulated as the Pycnodontid in Fig. 1.

An elevated pH seems to be as effective in suppressing decay as elevated salinity (Fig. 6). Calcite as bottom substrate has no buffering capacity with respect to pH because its solubility product (~3.3 * 10^–9^ mol^2^ kg^−2^) is too low. Consequently, all trials with CaCO_3_ as bottom substrate experienced acidification relative to seawater (pH ~ 8.1) to ca. 7 ± 0.5, one order of magnitude higher in H_3_O^+^ than seawater (Fig. 5). With sodium acetate and natron as bottom substrates (pH 8.6 and 11.2 respectively) no organic decay is noted. One reason could be that alkaline pH levels limit bacterial populations. All bacteria strive to keep their intracellular pH close to the neutral point^26^. Bacteria that live at neutral or slight acidity achieve this by pumping H^+^ from the intermembrane space to the matrix, in order to create an electrochemical potential necessary to synthesise ATP. By contrast, alkaliphilic bacteria like *Clostridium paradoxum* must lower intracellular pH in order to avoid degradation of proteins, and they achieve this by creating Na^+^ gradients^27–29^. That process is less energy efficient than H^+^ pumping. Furthermore, alkaliphilic bacteria metabolise sugars instead of amino acids. Possible consequences of these factors are that fish carcasses exposed to high pH decompose more slowly than at neutral or slightly acidic pH levels because absolute abundances of bacteria are limited. There is, however, a caveat to this interpretation: solutions in equilibrium with acetate and natron (cf. Fig. 3) have similarly high ionic strengths as hypersaline solutions with >10 wt.% NaCl equiv., so we cannot discriminate with confidence between the conserving effects of high salinity and high pH.

Redox conditions do not seem to have a major influence on decay rates. We have not noticed that anoxia around - 100 mV slows down decay significantly compared to more oxidised conditions^30^. That is expected. All bacterial strands that inhabit the intestines of fish are anaerobic or facultatively anaerobic. They are the first bacteria to begin decomposition of the carcass after a fish died, and they continue to do so for as long as a fish is not ruptured and exposed to the outside environment. Nonetheless, anoxia is important in fossilisation by keeping at bay scavengers.

### Application to important Konservat-Lagerstätten

We now apply our results to three Konservat-Lagerstätten where fish are prominent fossils. We decipher from the sedimentary records of the deposits environmental conditions favorable for fossilization. The fossil deposits we analyse are Eichstätt-Solnhofen, Green River, and Messel.

The Eichstätt-Solnhofen deposits of the Frankonian Alb are within an epicontinental, upper Tithonian (150 Ma) platform carbonate sequence deposited on the Helvetic shelf north of the Penninic ocean. Shallow reefs alternated laterally with restricted lagoons. The latter are filled with extremely fine-grained plattenkalks or lithographic limestones^31^. Fossil preservation is outstanding. Fossils include the famous *Archaeopteryx*, many species of pterosaurs, and a plethora of fish^32^. Many fish fossils are well articulated, and some even preserved original scale colouring^33^. In the Upper Jurassic the Solnhofen archipelago was situated at ca. 30°N in a semi-arid climate belt. The distribution of land and sea at that time - Laurasia in the northwest, Gondwana in the southwest, and the Tethys and Penninic oceans toward the southeast - suggests a monsoon-type climate, with wet onshore southeasterly winds in summer and dry offshore westerly winds in winter^34^. During winter times evaporation could have exceeded precipitation. Viohl^35^ assumes that many the Solnhofen-Eichstätt lagoons developed seasonal salinity stratifications where normal-salinity, oxygenated surface waters thriving with life overlay deep, hypersaline, reduced bottom waters. Fish that died at water depths where sinking was possible (Fig. 2) could have sunk into hypersaline bottom waters where decay was slow. Unfortunately though, salinities of the Solnhofen basins are poorly constrained. Seilacher *et al*.^36^ proposed that *post-mortem* contortions of vertebral columns of fish, quite common in Solnhofen, occurred when fish carcasses were dehydrated by hypersaline brines. Viohl^35^ reported what he thought were pseudomorphs of calcite after gypsum, suggesting salinities were above 11 wt.% NaCl equiv., but until primary gypsum is identified this proposition remains speculative. We can say though that the good conservation of fish carcasses in hypersaline brines does support Viohl’s^35^ analysis.

The Green River Konservat-Lagerstätte in Wyoming is one of the best fossil fish sites worldwide^37^. In the Eocene, the Green River lakes formed a playa-lake complex^38^ where fresh water periods alternated with highly alkaline, halite-saturated waters. Today, the lake sediments host the largest deposits of trona Na_2_CO_3_*NaHCO_3_*2H_2_O worldwide, a mineral that imposes at saturation a pH of >11. Eugster^39^ noted similarities with alkaline lakes of the East African Rift system that are also fossil fish bearing^40^. Grande^37^ implicated for the fish mortality events algal blooms or overturns of H_2_S-bearing hypolimnia but no convincing theories exist why the Green River fish are so well preserved. We propose that high salinities combined with high pH values may have played a key role for preservation.

The waters of the Eocene Konservat-Lagerstätte Messel may have been unusually alkaline as well. Messel was a crater lake^41^ whose waters must have equilibrated with alkaline basaltic tuffs. The hydrolysis of pyroclastic materials like feldspar and basaltic glass shards can impose on water high alkalinities and elevated pH values as high as 10 (ref. ^42^). In many fossiliferous horizons of the Messel stratigraphy fossils are encrustated by siderite FeCO_3_, a mineral that affords highly reduced conditions within the Fe^2+^,aq or Fe(OH)^+^,aq stability. Siderite is also a good pH sensor. At ambient CO_2_ partial pressure and an Fe^2+^,aq content of around 5 ppm, the formation of siderite in water requires a pH of >10 (ref. ^18^). It is possible that we owe the fish fossils in Messel to a highly alkaline pH.

## Conclusion

We show that success or failure in the fossilisation of fish is decided soon after a fish dies. Pressure must be high enough that a fish carcass may sink to the bottom sediment. A low redox state *per se* does not seem to delay soft tissue decay but anoxia may be essential in keeping at bay scavengers.

The parameters most effective in early fossilisation are a high salinity and an alkaline pH. When the salinity is >10 wt.% NaCl equiv. or the pH in the alkaline region, bacterial attack on soft tissue is greatly retarded, and a carcass can rest on the sediment-water interface for many weeks to months without decomposition until it is buried by sediment. The good preservation of the experimental proto-fossil in Fig. 8 is not meant to imply that fossilisation is complete after a few weeks. Many reactions will ensue, including the phosphatisation of bones^5^, the lithification by bacteria^43^, the pseudomorphic replacement of organic tissue by inorganic materials^44–46^, and the re-organisation of organic molecules to more durable compounds^47,48^. For successful fossilisation of fish it is the initial conditions that matter. They determine if a carcass rots, if it is consumed by scavengers, or if after millions of years it re-emerges in the geological record as a fossil.
